# Reasons for (non)compliance with intervention following identification of ‘high-risk’ status in the NHS Health Check programme

**DOI:** 10.1093/pubmed/fdu066

**Published:** 2014-09-18

**Authors:** R.J. McNaughton, J. Shucksmith

**Affiliations:** 1 School of Health and Care, Health and Social Care Institute, Teesside University, Middlesbrough TS1 3BA, UK; 2 Fuse (UKCRC Centre for Translational Research in Public Health), Newcastle University, Newcastle-upon-Tyne NE2 4AX, UK

**Keywords:** public health, screening, health services

## Abstract

**Background:**

The Department of Health introduced a risk assessment, management and reduction programme, NHS Health Checks, which aimed to reduce premature morbidity and mortality from cardiovascular diseases for those aged 40–74. Those identified as at increased risk of CVD are offered prophylactic medication and lifestyle advice to reduce their risk. Health gains will only be achieved if patients are compliant with advice/intervention however. This study sought to understand factors that influenced adherence to medication and advice in ‘high-risk’ patients.

**Methods:**

Qualitative data were collected through 29 semi-structured interviews with a purposive sample of individuals who had been identified as at high-risk of CVD. Participants had been offered lifestyle advice, lipid lowering medications and attended at least one annual review.

**Results:**

Findings explore the challenges and experiences confronting ‘high-risk’ individuals when making decisions about engaging with intervention. Key findings explore: statin adherence, as well as adherence to advice about diet, physical activity, alcohol consumption and smoking cessation.

**Conclusions:**

Attention needs to be paid to the way prophylactic medications are prescribed and explained to high-risk patients. Consistent provision of tailored lifestyle advice and access to appropriate services could facilitate sustained changes to factors that increase CVD risk.

## Background

It is widely believed that the majority of deaths that are attributed to cardiovascular diseases (CVD) could be prevented through the early identification of risk factors (for example, underlying physiological conditions such as hypertension, dyslipidaemia, diabetes and chronic kidney disease) and through the facilitation of lifestyle changes.^1–3^ It is said that identification of these risk factors and promotion of lifestyle changes to diet, physical activity, smoking and alcohol consumption could achieve a large reduction in mortality and morbidity associated with CVD^1,4^ and reduce the economic burden experienced by the National Health Service (NHS).^5–7^ However, the focus on so-called lifestyle factors tends to personalize the issues and frame them in terms solely of individual responsibility, deflecting attention from the social determinants of health. We know that in England, the burden of CVD is felt disproportionately in disadvantaged communities and therefore owes much to structural conditions of class and poverty.^8^ Mortality attributed to CVD has been falling for the population as a whole by ∼6% per year; however, this reduction has been experienced differently between socioeconomic groups, meaning that whilst overall rates fall, health inequalities are increasing.^9^ A major challenge for NHS Health Check (NHSHC) is therefore to reduce death and illness from CVD overall without actually increasing inequalities.

In the UK, the Department of Health (DH) has responded to this situation by developing a national CVD risk assessment, management and reduction programme: the NHSHC^4,10^ which was launched in 2009. The NHSHC aimed to offer CVD risk assessment to the entire population of 40–74 year olds in England and Wales within the first 5 years of the programme's implementation (by 2013).^4,10^ However, this aspirational target has yet to be achieved. Individuals who do not have pre-existing CVD or diabetes are invited to attend an appointment with their primary care practitioner to undergo an assessment of their risk of suffering a cardiovascular event in the next 10 years. This assessment includes the collection of anthropometric measurements, noting of family medical history and cholesterol testing. A calculation is then performed to assess the individual's CVD risk, and the outcome expressed as a percentage chance of suffering an adverse event in the next 10 years. Those individuals identified as being at 20% risk or higher are classed as ‘high risk’ and are therefore eligible for lifestyle advice and intervention with prophylactic medication (commonly statins) to reduce and manage their CVD risk.^10^

Initially, the commissioning of the NHSHC at the local level was the responsibility of Primary Care Trusts (PCTs). However, with the 2013 restructuring of the NHS, this responsibility was passed to Local Government^11,12^ with support from Public Health England.^13^ PCTs were originally given scope to develop the NHSHC programme in a way that suited the needs of their local population, whilst being mindful of the need to provide equity of access and avoid increasing health inequalities.^10^

In the study area NHSHCs were offered primarily through general practitioner (GP) surgeries, but also through community pharmacies^14^ (subsequently decommissioned) and in workplaces and other community settings, in an attempt to provide equity of access for the local population.

Under the current (national and local) guidance,^4^ once an individual has received an NHSHC and has been identified as at high risk of CVD (>20%) they are offered lifestyle advice, and in many cases will also be offered a lipid lowering medication (statin) for the purposes of prevention. In order to be successful the NHSHC must not only identify those at risk and offer intervention, it must also engage—and then sustain engagement—of the individual to comply with the intervention. Individuals must not only accept their invitation for assessment but also understand why they have been identified as at risk, understand advice given to them and adhere to any interventions offered over the long term. Without this adherence to the programme, the putative gains to the NHS from preventive^15^ action are unlikely to be realized.

This study reports on findings from a qualitative study that sought to understand patients' experiences of, and compliance with, forms of intervention offered as part of the NHSHC.

## Methods

Data described in this study were collected through 29 semi-structured interviews with patients who had received an NHSHC between 2009 and 2012. Participants were recruited through five GP practices located across four PCT areas in the North East of England, after they had attended an annual review. Those patients who agreed in principle to take part were provided with written information about the project to facilitate informed consent.

All participants had received an NHSHC, had been identified as at high risk (>20% of having an adverse cardiovascular event in the next 10 years), had received lifestyle advice and/or been prescribed a statin and had attended at least one annual review. These participants were chosen due to their experience of the NHSHC programme and because they had already had the opportunity to initiate and sustain lifestyle changes/medication regimens over a period of at least 1 year. Interviews were normally conducted in the participant's own home on a one-to-one basis, with the exception of three interviews where the participant's spouse was also present.

Interviews were conducted using a semi-structured interview schedule to facilitate frank and open discussions about the NHSHC and engagement with lifestyle advice and medical intervention. The interview schedule was developed using Normalisation Process Theory^16–19^ as a framework to sensitize the researchers to the process of implementing, embedding and integrating new practices into everyday life.

Interviews were, with the permission of the participants, recorded and transcribed verbatim. Data were anonymized and subjected to a six-stage thematic analysis.^20^ Initially, the researchers undertook a familiarization stage of analysis which led to the identification of initial codes. These codes were then applied to the whole dataset to enable the collation of codes into preliminary themes. These themes were reviewed by both researchers to produce a revised set of themes that were named and finalized. In the final stage quotations were selected which illustrated the thematic framework that had been generated. NVivo 9 was used to facilitate data management during the analysis stage.

The project proposal was scrutinized and approved by Teesside University School of Health and Social Care Research Ethics and Governance Committee.

## Results

Responses presented in this study are from 19 males and 10 females who were aged between 53 and 76 years old at the time of interview (see Table 1). During interviews participants were asked to recall if they had been offered lifestyle advice and access to services and if they had made changes to their lifestyle based on the advice received. Participants were also asked to recall if they had been offered a statin as a result of their risk assessment and, if they had, then initiated and continued treatment. An overview of the number of responses about each topic can be found in Table 2.

**Table 1:** Demographic characteristics of sample (sex, age, IMD quintile)

Sex	Male	Female				
No. participants	19	10				
Age in years	51–55	56–60	61–65	66–70	71–74	75+
No. participants	1	4	7	9	7	1
IMD (2010) quintile	Quintile 1 (least deprived)	Quintile 2	Quintile 3	Quintile 4	Quintile 5 (most deprived)	
No. participants	13	7	5	3	1

**Table 2:** Breakdown of sample by medication status and adherence to lifestyle change

**n* = 29*	*Not discussed at health check*	*Discussed: no changes made*	*Discontinued treatment*	*Successful change*
Statin medication	2	4 (statin refused at HC)	5	18
Dietary change	4	9	—	16
Increased physical activity	19	8	—	2
Reduced alcohol consumption	24	4	—	1
Smoking cessation	10 (non-smokers) 15 (ex-smokers)	3	—	1 (quit)

### Statin adherence

All participants were asymptomatic before attending their Health Check; they had been invited for assessment, not sought it out. The majority (*n* = 27) were offered a statin for the purposes of prevention; only two participants had not been offered a statin as a result of their assessment.

Of the 27 participants who had been offered a statin, four had refused outright. However, 23 had initiated statin treatment. During the course of the first year, 5 of the 23 had discontinued taking the statin permanently.

Whilst 18 participants had continued to take a statin for at least 1 year, post-risk assessment, this sustained compliance was due, in part, to a number of individuals being afforded the opportunity to reconfigure their medications. Side effects that were attributed to statin treatment were the main reason for wanting to discontinue the initially prescribed treatment. Many participants recounted the side effects they had experienced, which ranged in severity from mild, tolerable side effects (e.g. gastric discomfort) to symptoms that were really quite debilitating in some cases (e.g. severe muscular pains and heaviness of limbs). Upon experiencing side effects which participants attributed to the commencement of statin treatment, a number of them approached their GP or practice nurse to discuss the side effects. The majority of these participants were offered the opportunity to swap from the brand of statin they were currently taking to a new brand, in the hope that they would tolerate the medication better. In all of these cases participants noted that the side effects had disappeared and that they were happy to continue taking the new brand of medication as long as they were able to tolerate it. The opportunity to reconfigure medications was not the same in all GP practices. When some participants had returned to their practice to discuss the side effects they were experiencing, they had been turned away and told that their current brand was the only one that could be offered—in these cases all of the participants discontinued using statin. It was also reported that participants had discontinued statin treatment without returning to their practice due to the side effects they had suffered.

Testing for high cholesterol and trying to treat it through making dietary changes or by taking statin medication is a familiar concept in the UK, and it is often discussed in the national press and media. The topic of cholesterol management was often discussed in interview and many participants highlighted that they had been advised to take a drug that they understood was being promoted for cholesterol reduction for a new purpose—prevention, rather than cure. Statins were, in the eyes of participants, now being offered regardless of cholesterol levels or whether an individual's cholesterol was inside or outside the current recommended thresholds. This apparent lack of regard for prescription according to measured cholesterol levels caused confusion and anxiety. New concepts such as high-density lipoprotein level (HDL/‘good cholesterol’) and low-density lipoprotein level (LDL/‘bad cholesterol’) were being introduced into discussion in consultations as a way of encouraging people to commence statin treatment. However, this only served to muddy people's understanding of concepts they had previously thought they had a grasp on (Box 1).

Box 1 **Statin adherence**

They're fine. I mean I take it before I go to bed at night but, [laughs] it's when you get to bed…it bubbles in here [pats tummy]… I get wind, terrible wind, just bubbling away in here. Seemingly that's a side effect of them (P11, Female, taking statin).

 Well they [legs] stiffened up. It felt as though you'd been stood in a bucket of concrete! And, I got diarrhoea and felt sick and had a fuzzy head with them. They said [GP staff] ‘well you usually get this over the first three months’. It didn't ease off and I thought [to myself] ‘well, I felt perfectly well before I went on these; I'd like to come off them’ (P13, Male, taking statin).

 I went in [to the GP practice] and got them [statin] and she gave me a supply. I started taking them and all [of the] muscles in my back started jumping around like that [gestures with hands]. [I thought] ‘I can't go on like this’ and so I rang up [GP practice] and said ‘I'm not taking them’ (P19, Male, discontinued statin).

 Interviewer: So if you had some sort of ‘event’ like a heart attack or a stroke, you would be happy to take them [statin]? Respondent: Yes, because then it could be lifesaving! But I'm not too sure about [taking them] just for prevention? I mean there's no way that I would go and have a double breast operation, removal, just because I am at high risk of breast cancer—I just wouldn't do it (P5, Female, refused statin).

 I was aware of my cholesterol, and have been for over 15 years…[it is always] about 3.5. That's about what my cholesterol is, you know, always has been. She [the nurse] said ‘yeah, but your bad cholesterol is higher than your good cholesterol’. I thought ‘what's she on?’ ‘Where's she coming from?’ ‘Bad’ cholesterol, ‘good’ cholesterol…never heard of it! (P15, Male, taking statin).

### Advice about diet

Recollection of dietary advice in the NHSHC consultation was high. Twenty-five participants recalled being offered advice about their diet; 16 participants stated that they had made some changes to their eating habits based on that advice; 9 participants stated that they had made no changes to their eating habits; 4 participants could not recall being offered any advice.

Participants were happy to discuss diet as part of their NHSHC, but how these discussions were broached had an impact on the participant's experience, especially when these discussions were framed in the context of losing weight. Identification of individuals as obese had the potential to leave them with negative feelings about the whole assessment.

For many participants, making small and sustainable changes to their diet by consuming less salt and fat was achievable, as long as it did not cause too much disruption to their daily routines. A number of older participants felt that making changes to their lifestyle was unnecessary at their age, and the provision of healthy eating information was not always accepted and was branded too generic. There was a call for dietary advice that was CVD risk reduction focussed, as opposed to the normal information that could be picked up from any doctor's waiting room.

Like the discussions about thresholds for cholesterol and how they seemed to alter frequently, people showed reluctance to make changes to their lifestyle, noting that any guidance they were given was likely to be subject to change. Many cited the consumption of eggs as an example, stating that previous guidance about healthy eating suggested that the consumption of eggs should be restricted; then the reverse was promoted—eggs were recommended as part of a healthy balanced diet (Box 2).

Box 2 **Advice about diet**

I must admit, when she [the nurse] said to me that I was ‘obese’ I was devastated…it really shocked me, didn't it? [husband nods]. I couldn't get over it. I was really like ‘I can't believe I'm obese’ (P3, Female, discontinued statin).

 You know, I'm 72 this year and you think to yourself ‘oh crumbs, I'm done!’ I am eating everything right. We both [he and his wife] have a drink, we like a drink, but if my main doctor says ‘just go and live your life, that's what I do (P19, Male, discontinued statin).

 She [ the nurse] gave me some paraphernalia [about healthy eating] but it was a waste of money giving me that, because I just didn't bother with it…I'm quite happy [the way I am] (P17, Male, taking statin).

 They say eat margarine, then butter is better for you, [then] don't eat margarine. That's what I'm saying, I don't take any notice of all of these ‘you must eat this’ because two years later they are saying they are good for you…at one time eggs was bad for you, do you remember? Then they decided, no, eggs are good for you (P27, Male, discontinued statin).

### Advice about physical activity

When asked to recall advice that had been offered with regard to physical activity levels, 10 participants recalled receiving advice and 2 went on to take action and increase their personal physical activity levels. Eight participants did not make any changes to physical activity levels based on the advice they were given; 19 participants could not recall being offered any advice about physical activity levels. No one recalled being offered a referral to physical activity programmes either ‘in house’ at the GP practice or in a community setting.

Many interviewees were already fairly active before they attended their NHSHC. Many enjoyed swimming, walking and group activities such as bowls on a regular basis. However, many found that due to their advancing age, they were restricted in what activities they could now undertake. These people preferred to incorporate physical activity into their daily lives, through activities such as gardening, which in itself was often challenging, especially when they had co-morbidities such as arthritis, and they were of the view that any physical activity intervention suggested to them would have to take these impairments into account if it were to be acceptable (Box 3).

Box 3 **Advice about physical activity**

On average, I walk four miles a day. I go on the hills [and] climb (P27, Male, discontinued statin).

 I mean I bowl twice a week; green bowling, lawn bowling, and in the wintertime it's once a week because we do carpet bowls and I walk up and down the village every day (P11, Female, taking statin).

 I don't feel old, but then my body is telling me that I am. I tried running, because I used to be cross country at school. But now I can't run 5 yards, and it's my body telling me that I'm old. I only feel 19 but then when I try to do something, I know I'm not (P8, Male, taking statin).

 I can garden and that, but I have got arthritis and so I battle a bit. I have got a thing that I kneel on to get up and down (P28, Female, taking statin).

### Advice about alcohol consumption

Discussions about alcohol consumption were recalled less frequently. When asked to recall if they had been offered advice about alcohol consumption, five participants could recall being offered advice. One participant made changes to his/her alcohol consumption based on that advice, whilst 24 participants could not recall being offered any advice about alcohol consumption.

For the majority of participants, alcohol consumption was not discussed but they felt that their alcohol consumption was within guideline amounts, and for those who had discussed their consumption with the nurse at the HHC, this had been reiterated during discussions. There was only a small minority who had decided to reject advice from the nurse (Box 4).

Box 4 **Advice about alcohol consumption**

She said I was quite in the limit of what I drank. You know, because we have wine on a Sunday and a couple of brandies sometimes. Not two on a night, but one on a night, a couple of times a week (P11, Female, taking statin).

 [the nurse said] ‘I think you should have two nights off. ‘Oooooh’ I said ‘which nights would they be [nurse's name]? [The nurse said] ‘Monday and Sunday?’ [I said] ‘No, after line dancing it's my cricket club night!’ (P9, Female, refused statin).

### Advice about smoking cessation

The majority of participants were either non-smokers (*n* = 10) or ex-smokers (*n* = 15) so had not been offered smoking cessation advice. Four participants were smokers at the time of their risk assessment and were offered smoking cessation. One of these smokers went on to quit smoking as a result of the smoking cessation offered during the risk assessment. Amongst those who had discussed smoking cessation and declined the invitation, there was a feeling that it was being offered to them too late in their lives to make any difference to their health (Box 5).

Box 5 **Advice about smoking cessation**

…it's too late, yeah? It's far too late. I mean 67, say I live another 20 year, [that would make me] 87, which would be brilliant but it would take 20 year minimum for my lungs to clear, minimum (P15, Male, taking statin).

## Discussion

### Main findings of this study

All participants had engaged with the NHSHC programme insofar as they had attended their assessment, been offered advice and gone on to attend at least one annual review—making them already somewhat compliant with the programme. Nevertheless, the advice they were offered at the time of assessment and subsequent initiation and adherence to lifestyle changes and lipid lowering medications was highly variable within the sample.

Discussions about lifestyle changes were broached variably, with discussion about diet happening most frequently. Discussion about physical activity and alcohol consumption happened much less frequently and indicates an area for improvement within the programme. People were more receptive to making small but manageable changes to their lifestyle and they were open to the provision of information about how to reduce CVD risk through dietary changes. However, they were confused (and somewhat undermined) by the fact that guidance could be subject to change. When it came to the promotion of increased physical activity, people highlighted that there would need to be consideration of those who experienced co-morbidities often associated with ageing.

Side effects from statin were the main reason for discontinuation of drug treatment. How discussions about side effects were handled by health professionals had a great bearing on a participant's decision to try a different brand or to discontinue treatment on a permanent basis. Participants did not distinguish between the effect of statin to lower cholesterol or its promoted overall preventative effects.

The promised benefits from this universal intervention are theoretical rather than proven at this stage in the production of empirical evidence. Predicted gains from modelling assume high levels of compliance after testing and identification of ‘high-risk’ patients. This qualitative study indicates first a level of variability amongst health-care professionals in raising for discussion, post-test, different aspects of the available drug and lifestyle treatments. Secondly, it indicates that patients experiencing side effects from statins are more likely to remain adherent if GPs are willing to listen to their concerns and review medication. Thirdly, the study emphasizes that for patients deemed to be at high risk the Health Check is not so much an event as the start of a process of adaptation to a new lifestyle which requires far more personalized and tailored advice on diet and activity suitable for people in later age and possibly with existing co-morbidities.

### What is already known on this topic?

The DH's modelling exercise estimated that, if successfully implemented, the NHSHC could be cost-effective.^15^ However, early real-world findings suggest that uptake is lower than expected^21,22^ and that national coverage stands at 8%, well below the DH's expected coverage of 18% by 2012.^23^ In 2012 NHSHCs came under criticism as the intervention had been rolled out based upon theoretical modelling and not on evidence from randomized controlled trials,^24^ leading to calls for the abandonment of the NHSHC programme until the evidence base for effectiveness is stronger.^25^ Rebuttals have been played out in the *BMJ*, defending the universal programme,^26^ but with some authors advocating the development of a targeted programme,^27,28^ rather than the current universal approach.

Socioeconomic status, ethnicity and gender have all been positively associated with increased risk of CVD and it is known that many interventions can actually increase health inequalities.^9,29^ It has been suggested that, rather than taking a universal approach to identifying and treating risk factors (which may increase inequalities even more as a consequence of differential uptake), a population approach (focusing on deprivation and population wide policies, e.g. tobacco control or making healthy food choices affordable) to promote cardiovascular health would be both more effective and more cost-effective.^30–34^

Although statins have been found to be effective in the primary prevention of CVD,^35^ previous studies have found that overall adherence to treatment is low, with only half of those prescribed statin taking them on a daily basis.^36^ Patients receiving treatment with statin for primary prevention, as opposed to secondary prevention, are more likely to discontinue treatment.^37,38^ Side effects are often cited as the reason for discontinuation of statin treatment,^39^ as was found in our study.

### What this study adds

Much of the published work on NHSHCs so far has focussed on implementation, uptake and coverage of the UK programme. The focus in these studies has been on initial assessments and conversion of invitations into assessments for example. Less attention has been paid to the longer term adherence amongst those individuals who have been identified as at high risk of CVD. It is important, especially in a time of depleted budgets, to ensure that any intervention is offered to the right people, at the right time and most importantly that those people are accepting of and compliant with the intervention. If this does not happen, the NHSHC programme cannot realize its full potential.

This study suggests that attention needs to be paid to a more sophisticated prescription of prophylactic medications to reduce CVD risk and also to better explanation of their virtues and value to patients. The study also suggests the need for provision of more tailored lifestyle advice and access to appropriate services to facilitate sustained changes to factors that could increase CVD risk.

### Limitations of this study

Findings in this study are derived from a relatively small number of interviews with individuals identified as at high risk. All were White British (reflecting the demographic balance in the catchment studied however) and the majority were residents in the least deprived quintiles of Tees (Table 2). With qualitative studies generalizability achieved through having a large, representative sample, is not the aim. Rather, we seek insights developed through looking at issues in great depth, but further studies of compliance in patients from more deprived quintiles or from populations with different ethnicity are clearly called for.

Participants were asked to recall what lifestyle advice and medical intervention had been offered to them some time ago. The lapse of at least 1 year (patients were contacted after their first annual review) may have affected the accuracy of their recall around what was offered in terms of lifestyle advice/intervention, but their current behaviour is clearly determined by their memory and understanding of that encounter.

Findings were drawn from interviews with people who were already compliant with some aspects of the NHSHC programme. We have no data from people who:

1. failed to attend their risk assessment (refusers) or
2. attended their risk assessment but did not attend an annual review (dropouts).

Further qualitative research is needed to understand the needs and experiences of these two groups and should include representation from ethnically diverse populations.

## Authors’ contributions

R.M. contributed to developing the bid for funding, the design of the study, recruited participants, carried out fieldwork, analysed the data and wrote the article. J.S. secured funding for the project, designed the study, analysed data, and commented on and revised drafts of the article.

## Funding

This work was supported and commissioned by a collaboration of four North East Primary Care Trusts. The views expressed in this article are those of the authors and not necessarily those of the funding body.
